# Mitochondria-targeted ROS scavenger JP4-039 improves cardiac function in a post-myocardial infarction animal model and induces angiogenesis ***in vitro***

**DOI:** 10.1371/journal.pone.0320703

**Published:** 2025-04-24

**Authors:** Rayane Brinck Teixeira, Jane H. Albro, Mohamed Sabra, Taslova Abedin, Aja N. Tucker, Raj Sidharth, Frank W. Sellke, Peter Wipf, M. Ruhul Abid

**Affiliations:** 1 Department of Surgery, Division of Cardiothoracic Surgery, Cardiovascular Research Center, Rhode Island Hospital, Warren Alpert Medical School of Brown University, Providence, Rhode Island, United States of America; 2 Department of Chemistry, University of Pittsburgh, Pittsburgh, Pennsylvania, United States of America; 3 Department of Pharmaceutical Sciences, University of Pittsburgh, Pittsburgh, Pennsylvania, United States of America; 4 Department of Bioengineering, University of Pittsburgh, Pittsburgh, Pennsylvania, United States of America; Jhargram Raj College, INDIA

## Abstract

**Background:**

This study aimed at evaluating the effects of JP4-039, a mitochondria-specific reactive oxygen species (mito-ROS) scavenger, on coronary angiogenesis and cardiac function in a post-myocardial infarction (MI) animal model.

**Methods:**

Mice underwent ligation of the left anterior descending (LAD) artery to induce MI and received intraperitoneal (i.p.) injections of JP4-039 or vehicle (n=8 animals/group) three times/week for four weeks. Echocardiography for cardiac function and immunohistochemistry for Infarction area and capillary density were carried out. Angiogenic potential of endothelial cells (EC) was assessed by *ex vivo* tube formation using mouse heart EC (MHEC) and by aortic and atrial sprouting. Western blots were conducted using mouse cardiac tissue and lysates from HCAECs that were treated with or without JP4-039.

**Results:**

Cardiac function including ejection fraction, fractional shortening, and fractional area change were improved significantly in JP4-039-treated animals compared to the vehicle group. JP4-039-treated hearts demonstrated significant reduction in infarction size and increased capillary density in the ischemic area. These findings were consistent with increased *ex vivo* endothelial sprouting of the aortae and atrial tissue from the mice treated with JP4-039. Western blots using cardiac tissue lysates from JP4-039-treated animals showed decrease in phosphorylation of AMPKα at the Threonine 172, suggesting a plausible increase in the ATP:AMP ratio. Interestingly, JP4-039 increased expression of mitochondrial complexes I and IV and increased ATP synthesis in EC.

**Conclusions:**

JP4-039-mediated reduction in mito-ROS results in significantly increased coronary vascular density in ischemic myocardium, improved ATP synthesis, and recovery of post-MI cardiac function. Together, these results suggest that nitroxide nanodrug-mediated reduction in mito-ROS may help recover post-MI cardiac function.

## Introduction

Ischemic heart disease (IHD) is the leading cause of death globally, accountable for 8.9 million deaths in 2019 [1]. Acute myocardial infarction (MI), often referred to as heart attack, is caused by a sudden cessation of blood flow to a given myocardial territory. This sudden cessation in delivery of oxygen and nutrients leads to widespread impairment of cellular processes such as redox homeostasis that follows dysregulation of reactive oxygen species (ROS) and culminates in profound vascular and cardiac tissue injury [2,3]. During ischemia, hypoxia leads to a number of mitochondrial dysfunctions including impaired mitochondrial electron transport chain (ETC) activity, altered ion homeostasis, shifting of metabolic substrate utilization, and excessive ROS formation, leading to mitochondrial dysfunction [4]. This cascade can result in energy deprivation due to ATP depletion, aberrant cellular calcium homeostasis, and programmed cell death through the release of cytochrome *c* into the cytosolic compartment and activation of caspases. Over time, mitochondrial dysfunction with excess ROS contributes to endothelial dysfunction, vascular smooth muscle pathology, myofibrillar disruption, and altered cell differentiation, which lead to cardiac remodeling, fibrosis, and eventual heart failure [4,5]. Given that excessive ROS formation is a common underlying mechanism of endothelial cell (EC), cardiomyocyte, and mitochondrial dysfunction, extensive research has been performed on the utility of antioxidants to prevent and/or treat cardiovascular disease (CVD) [6–9]. It is now recognized that global antioxidant therapies lack measurable benefits in overall patient outcomes and may even increase cardiovascular mortality [10].

Although therapies for IHD and heart failure have improved and can protect cardiac function by reducing energy demand, impaired energy supply during ischemia remains a major challenge. Increasing energy supply has been a goal of future therapeutics to preserve cardiac function in CVD [4]. Since mitochondria are an important source of energy, restoration of mitochondrial function has been a target of novel therapies, including mitochondria-targeted antioxidants. Several studies have examined the acute effects of mitochondria-targeted antioxidants, such as XJB-5–131, SS31, and MitoQ, in non-survival models of MI and I/R [11–14]. The current *in vivo* study investigated a mitochondria-targeted antioxidant JP4–039, which is a mitochondria-enriched nitroxide-compound with significant ROS and electron scavenging properties through its nitroxide radical moiety, 4‐amino‐TEMPO [15,16]. JP4–039’s mitochondrial localization and abrogation of oxidative stress-mediated events in the mitochondrial membrane inhibit cardiolipin oxidation (CL_ox_), CL_ox_ binding to cytochrome c, and leakage of cytochrome c into the cytoplasm, supporting JP4–039’s role in radiation mitigation and protection [17]. JP4-039 was initially studied as an agent that protected against total body irradiation, until its mitochondrial ROS scavenging properties prompted interest in other disciplines [18–20]. In particular, our laboratory has previously described the effects of delivering JP4-039 released from electrospun polymeric nanofibrous scaffolds on angiogenesis of human coronary artery ECs (HCAEC) [18]. Released JP4-039 was shown to preserve the active antioxidant moiety and improve tube formation and migration of HCAEC [18]. However, the effects of JP4-039 on cardiac function using relevant models of heart disease, such as in myocardial ischemia (MI), have not been evaluated. Thus, the current study aimed at evaluating the *in vivo* effects of JP4-039 in an animal model of MI by assessing post-MI coronary angiogenesis, infarct size, and cardiac function recovery will have significant impact on cardiovascular disease research.

## Methods

Please see the S1 Table in the supplemental materials.

### Ethical statement

This study was approved by the lifespan IACUC under the protocol number 5019-19.

### Experimental design

This study contains data from *in vitro*, *in vivo*, and *ex vivo* studies. A summary of this study’s experimental approach is available in S1 Fig.

### In vitro study

Our *in vitro* study was conducted using primary adherent culture of commercially available HCAECs and of transgenic mouse heart endothelial cells (MHEC) overexpressing NOX2 (NOX2-OE), isolated in our laboratory (after 4.8 months of NOX-2 OE in vivo). HCAEC and NOX2-OE MHEC were treated *in vitro* with either vehicle or JP4-039 at a final concentration of 5 µM for 1.5 and 3 hours. Mitochondrial ROS (Mito-ROS) production, mitochondrial redox state, and angiogenesis were evaluated by MitoSOX, Mito-roGFP, and tube formation assay, respectively.

### *In vivo* study

Our *in vivo* study was conducted in wild type FVB mice aged 6 ± 1 week old, consisting of 50% male and 50% female mice. This study used FVB mice to allow for correlation with the results obtained in a similar study where decreased Mito-ROS was investigated in a transgenic model that has the same background [21]. The MI model in FVB mice was characterized by reporting echocardiogram and histological findings for model validation.

To have a record of the baseline cardiac function, all mice had the left ventricle function assessed through transthoracic echocardiography prior to starting the protocol. The protocol started at day 0, when mice were anesthetized and randomly subjected to SHAM (SHAM group) or LAD ligation surgery (groups vehicle and JP4-039). Mice received their first intraperitoneal (i.p.) injection of either vehicle (groups SHAM and vehicle) or JP4–039 (group JP4-039) immediately after closure and suture of their chest, and then allowed to regain consciousness before returning to their cages. Subsequently, mice were weighed and injected with their respective i.p. treatments (vehicle/JP4-039; 1 mg/ kg/ injection) three times per week for a total of four weeks. Injections were administered in the morning. LV function was assessed again at twenty-eight days after surgery, followed by euthanasia and tissue harvesting. Mice were sacrificed by anesthesia overdosing (intraperitoneal injection of 200 mg/kg ketamine and 20 mg/kg xylazine), followed by 5% isoflurane inhaled until cessation of breathing as per approved IACUC protocol # 5019-19. Heart was collected and divided into basal and mid-to-apex piece. The basal section was snap-frozen and later used for western blot of whole tissue lysate. The mid-to-apex piece was mounted in O.C.T compound and later used for histology and immunofluorescence to evaluate fibrosis, and capillary and arteriole densities.

### Ex vivo study

Following sacrificing, aorta was harvested and immediately processed ex-vivo for an aortic sprouting assay. In addition to aortic sprouting using aorta from infarcted mice after *in vivo* treatment, our ex vivo study evaluated atrial sprouting using atrial tissue from control FVB mice with *ex vivo* treatment with vehicle/ JP4-039.

## Methods used in the *in vitro* study

### Dilution of JP4-039 for cell culture

JP4-039 was first dissolved in pure 2,2,2-triﬂuoroethanol (TFE) and then diluted in Dulbecco’s phosphate buffered saline (DPBS) to a stock solution of 250 µM JP4-039 in 1% TFE in DPBS. The stock solution was then diluted to a final concentration of 5 µM JP4-039 in EGM2-MV media. Another solution containing the same amount of vehicle only (no JP4-039) was diluted in EGM2-MV media and used as control. For the *in vitro* study, we performed initial dose-response-curves using different concentrations of JP4-039 in HCAECs, and quantified mito-ROS content for each concentration. We found that 5 µM JP4-039 was consistently able to decrease mito-ROS significantly.

### Measurement of mitochondrial redox state using Mito-roGFP

Human coronary artery endothelial cells (HCAEC) were plated into a µ-dish^35mm, high^ glass bottom (ibidi GmbH, Germany) at a density of 15 × 10^3^ cells/well (passage 4). Cells were cultured in EGM2-MV media for 48 hours and then transfected with Mito-roGFP. Media was changed on the day following transfection. Two days after transfection, HCAEC were treated with vehicle or 5 µM JP4-039 for 1.5 hours, followed by live-cell ratiometric fluorescence imaging in an inverted microscope (Nikon ECLIPSE TE20000-U from Nikon Instruments Inc., NY, USA) [21–23].

### Measurement of mitochondrial ROS using MitoSOX reagent

HCAEC were plated into Costar 96-well clear bottom, black plates (Corning Incorporated, Corning, NY) at a density of 20 × 10^3^ cells/well in EGM2-MV media (passage 5). Cells were incubated in hypoxia condition. Hypoxia incubation was performed as previously described [24]. Cells were treated with Vehicle or JP4-039 and re-incubated in hypoxia for 1 hour and 30 minutes, then labeled with 5 µM MitoSOX^TM^ Red reagent in Hanks Balanced Salt Solution (HBSS) for 10 minutes (Thermo Fisher Scientific Inc., USA). For background control wells, the cells received the same treatments, except for the 10-minute incubation step, in which background control wells were incubated only with HBSS (no MitoSOX^TM^ was added). Total incubation time in hypoxia was 24 hours. Mito-ROS fluorescence was read at a BioTek SynergyMx microplate reader (Agilent, USA). Results were calculated as a ratio from the average background reading.

### Tube formation assay

Human coronary artery endothelial cells (HCAEC), or MHEC with prolonged overexpression of NOX2 were plated into a 15-well µ-slide Angiogenesis (ibidi GmbH, Gräfelfing, Germany) precoated with Cultrex® basement membrane extract (BME, R&D Systems, Minneapolis, MN) at a density of 10^4^ cells/well (passage 4), following manufacturer protocol [6]. Cells were diluted in the EGM2-MV media containing 5 uM JP4-039 for test wells or an equal volume of vehicle for vehicle wells prior to plating (total of 5 wells per group). Cells were kept in normoxia conditions inside an incubator at 5% CO_2_ and 37 °C and imaged after 3 hours using 4× magnification phase contrast in an inverted microscope (Eclipse Ts2, Nikon Instruments Inc., Melville, NY). On a separate assay, cells were kept under hypoxia conditions and imaged after 24 h using 4× magnification phase contrast in an Olympus CKX53 inverted microscope [24]. The number of tubes was measured using ImageJ software.

### Scratch assay

HCAECs, at passage 5, were plated in a 12-well plate (CytoOne) at 5 × 10^5^ cells/mL. Once plated, these cells were put in hypoxic conditions using a modular hypoxic chamber (Billups-Rothenberg). To create the hypoxic environment, the oxygen inside the chamber was replaced with 95% N2 and 5% CO2 at 20 L per min flow rate and allowed to flow for 7 minutes [24]. The chamber containing the cells was placed into an incubator kept at 37 °C overnight. Prior to the scratch, a JP4 stock solution was made by dissolving 0.01613 g of JP4 in 1 mL of TFE and diluted with 99 mL of PBS. The 5 µM JP4 working solution was made with 1 mL of stock solution and 49 mL of EGM-2MV cell media (Lonza, Walkersville, MD). The cells were washed with PBS before treated with either JP4 media or equal volume of released vehicle media. A 200-µL tip was used to make a transversal scratch down the middle of each well. Cell debris was washed away using either JP4 or vehicle media respectively and media was replaced in each well. The cells were kept under hypoxic conditions inside the 37 °C incubator and imaged at hour 0, 3, 6, and 24 at 10× magnification using the inverted microscope (Olympus CKX53) and scope camera (Olympus EP50). The area of each scratch was measured using Image J and the data reflects a percent decrease in area over time compared with the total area.

### Estimation of ATP by luminescence

Intracellular content of ATP was estimated by luminescence using the ATPlite 1step luminescence assay system (Perkin Elmer Inc., Shelton, CT) [25,26]. HCAEC were seeded in a 96-well black plate with clear bottom at a density of 20 × 10^3^ cells/well. Cells were allowed to grow overnight. After treatment with vehicle/JP4-039 for 3 hours, 100 µl of the ATPlite reagent was added to each well. The contents were then mixed in a plate shaker for two minutes at 700 rpm followed by luminescence measurement on a plate reader (SpectraMax M Series, Molecular Devices, San Jose, CA).

### Methods used in the *in vivo* study

#### Myocardial infarction and SHAM surgeries.

All animal studies were carried out under the approved IACUC protocol # 5019-19 of Brown University Health. MI was induced by a permanent ligating the left anterior descending coronary artery, as previously described [27,28]. For the SHAM surgery, all steps were followed similarly to the MI surgery, except that the suture was only passed around the artery without performing ligation [29]. Methods of anesthesia consisted of an intraperitoneal injection of Ketamine (100 mg/kg) supplemented with 4% isoflurane (inhaled through VetFlo system, Kent Scientific, Torrington, CT) to induce anesthesia, followed by 2–3% inhaled isoflurane delivered with oxygen via intratracheal tube by use of a MiniVent Ventilator for Mice (Model 845, Harvard Apparatus, Holliston, MA) during the entire surgical procedure. Absence of reflexes was confirmed by toe pinch and by checking eye blink reflex before starting the procedure. In order to alleviate suffering, post procedure analgesia consisted of a single subcutaneous injection of 1 mg/kg of a 72-h slow-release buprenorphine solution. After surgery, animals were allowed to recover in a clean cage placed on top of a 37 °C recirculating water system (HTP-1500 Heat Therapy Pump, Kent Scientific, Torrington, CT). Animal appearance was checked each morning and afternoon for three days following surgery by lab personnel and checked daily by a veterinarian technician. An additional cup containing crushed food moistened with water was added to the bottom of each cage to warrant nutrition and hydration for three days following surgery. Animals were sacrificed on post-operative day (POD) 28 using euthanasia procedure as approved by the IACUC protocol # 5019-19.

#### Echocardiography.

Echocardiography was performed on the left ventricle using the Vevo 2100 ultrasound system with an MS550 transducer (FUJIFILM VisualSonics, Inc., Bothell, WA). To induce anesthesia, each mouse was placed in a box with flowing oxygen and 4% isoflurane (inhaled through VetFlo system, Kent Scientific, Torrington, CT). Then each mouse was placed in a 37 °C heated examination table and received 2–3% inhaled isoflurane, delivered through a nose cone during the entire procedure. Cine loops were acquired at the short-axis view in both two-dimensional and M-mode at the apex, Mid-papillary, and basal levels [30]. Acquired images were used to measure ejection fraction (LVEF), fractional shortening (LVFS), heart rate (HR), cardiac output (CO), end systolic volume (LVESV), end diastolic volume (LVEDV), stroke volume (LVSV), end systolic diameter (LVESD), end diastolic diameter (LVEDD), systolic anterior wall thickness (LVSAWT), diastolic anterior wall thickness (LVDAWT), systolic posterior wall thickness (LVSPWT), diastolic posterior wall thickness (LVDPWT), fractional area change (LVFAC), end systolic area (LVESA), and end diastolic area (LVEDA). Images were analyzed using Vevo LAB 5.5.0 software [29,30].

#### Dilution of JP4-039 for the in vivo study.

JP4-039 powder was dissolved in pure DMSO at 1 mg/µl concentration with vigorous vortexing until completely dissolved. JP4-039 was then diluted in sunflower seed oil to a concentration of 0.01 mg/µl (stock solution). This stock solution was then further diluted in sunflower seed oil to a final volume of 100 µl and a final concentration of 1 mg/kg of body weight and administered via intraperitoneal (i.p.) injection. A vehicle solution was also prepared as described above, using only DMSO and sunflower seed oil (without JP4-039 powder) [11]. Injections were always administered in the morning within 30 minutes of drug dilution. For the in vivo study, JP4-039’s dosage was determined empirically. In prior *in vitro* studies performed in our lab, we found that JP4-039 achieved similar results then XJB-5–131 at half the concentration used (5uM JP4–039 versus 10 uM XJB-5–131). Escobales and collaborators had treated aged rats with XJB-5–131 at a dose of 3 mg/kg, via i.p. injections three times per week prior to I/R [11]. Considering prior results in our lab showed that JP4-039 was effective in HCAECs at half the dosage needed for XJB-5–131, a dosage of 1.5 mg/kg was calculated accordingly. Since we were injecting animals intraperitoneally three times per week for four weeks post-MI, we empirically chose to use 1 mg/kg instead of the estimated 1.5.

#### Masson’s Trichrome staining.

Heart cross sections were stained using a Masson’s Trichrome stain kit (from Polysciences Inc., Warrington, PA), following manufacturer protocol. Infarction area (Midline length) was quantified as previously described [31].

#### Heart immunofluorescence.

Frozen section immunofluorescence was conducted using CD31 (1:100) and SMA (1:100) antibodies. Anti-rat Alexa 488 (1:200) and anti-rabbit Alexa 594 (1:200) were used as secondary antibodies. Samples were processed following protocols from cell signaling.

#### Western blotting.

Western blotting was conducted to investigate protein expression in heart homogenates and HCAEC lysates. In brief, the basis of the heart was homogenized in 1x RIPA buffer solution containing 1% protease inhibitor cocktail (SIGMA catalog P8340), 1% phosphatase inhibitor cocktails 1 and 2 (SIGMA catalog P5726, P0044) in addition to 5 mM sodium fluoride and 1 mM sodium orthovanadate. Samples were homogenized with a tissue tearor (model 985370) at medium speed for 40 seconds. Total protein concentration was measured by a BCA assay kit (Thermo Fisher Scientific Inc., Carlsbad, CA). Electrophoresis was performed in the NuPAGE system by loading 35 µg (heart homogenates) or 10 µg (HCAEC lysates) of protein/sample. Proteins were subsequently transferred overnight into a nitrocellulose membrane. Protein expression of p-AMPKα Thr^172^ (cell signaling catalog 2535) and GAPDH (cell signaling catalog 97166) was imaged via chemiluminescence and densitometry was quantified using ImageJ [32]. For the OXPHOS western blotting, samples were loaded in the gel without prior heating and the gel was transferred overnight to a PVDF membrane. Membrane was incubated overnight with total OXPHOS rodent antibody cocktail (Abcam catalog ab110413) and vinculin (Thermo Fisher catalog PA5–29688), and imaged via chemiluminescence and densitometry was quantified using ImageJ [32].

### Methods used in the *ex vivo* study

#### Aortic and atrial sprouting ex vivo.

Aortic sprouting was done as previously described [33]. Mice were sacrificed by anesthesia overdosing (intraperitoneal injection of 200 mg/kg ketamine and 20 mg/kg xylazine), followed by 5% isoflurane inhaled until cessation of breathing as per approved IACUC protocol # 5019-19. Following harvesting 28 days post-MI, the aortas were collected, cleaned, cut into 1 mm thick rings, and plated in a 48-well plate on top of polymerized BME (reduced growth factor, R&D Systems), n=8 rings per mouse. Another layer of BME was added on top of each ring and polymerized. Aortic explants were cultured in EGM2-MV media and allowed to sprout for 6 days. Sprouts were imaged in an inverted microscope (Nikon ECLIPSE TE20000-U from Nikon Instruments Inc., NY, USA). Images from 5 days after plating were used for measurement of the outgrowth area on ImageJ [32]. The atrial sprouting was performed similarly to the aortic sprouting. However, the atrial tissue was collected from control (no intervention) mice. After plating the atrial explants were cultures in EGM2-MV media containing vehicle or JP4–039. Media was changed every 48 h with fresh treatments every time. Images from 6 days after plating were used for measurement of the atrial outgrowth area.

### Statistical analysis

Statistical analysis was conducted using GraphPad Prism 9 software. Data were normality-tested by Shapiro-Wilk. One-way ANOVA followed by Tukey’s multiple comparisons test were applied for normally distributed data. Nonparametric data were analyzed by Kruskal-Wallis followed by Dunn’s multiple comparisons test. When only two groups were compared, normal data were analyzed by Student’s t-test and nonparametric data were analyzed by Mann-Whitney test. Results with an alpha value inferior to 0.05 (p < 0.05) were accepted as significant. Parametric data is plot as bar graphs while nonparametric data is shown as violin plot. Graphs were prepared using GraphPad Prism 10.

## Results

### JP4-039 decreases mitochondrial ROS in endothelial cells

**Fig 1:**
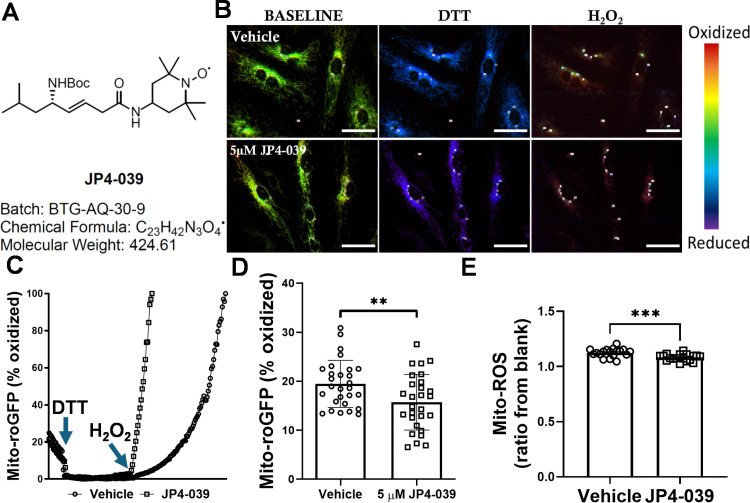
JP4-039 decreases mitochondrial ROS in human coronary artery endothelial cells *in vitro.* (A) Structure and batch number of the mitochondria-targeted ROS scavenger JP4-039 are shown. (B) Representative images of oxidative status measured using Mito-roGFP adenovirus construct based on HCAEC’s response to DTT and H_2_O_2_ using ratiometric analyses are shown. (C) Representative time-lapse curve showing % oxidation, and live response to DTT and H_2_O_2_ are plotted. (D) Baseline % oxidation measured in different regions of interest are shown. n=28 regions of interest/group (E) Mito-ROS fluorescence under hypoxia condition is shown. n=18 wells/group. **p<0.01, ***p<0.001. Results were analyzed by Shapiro-Wilk and Student’s t-test. Scale bar represents 50 micrometers.

In order to confirm the effects of JP4-039 (Fig 1A) on mito-ROS of endothelial cells, HCAEC were treated with JP4-039 and subject to measurement of ROS using mitochondria-specific ratiometric fluorescence (Fig 1B). To that end, HCAEC were transduced with Mito-roGFP, a mitochondria-targeted replication-deficient adenovirus construct that allows real-time measurement of the redox status in live cells via ratiometric fluorescence measurements [22,23]. JP4-039 was found to decrease the baseline redox status of HCAEC by 19% in comparison with the vehicle (19.5±0.9% basal oxidation in vehicle vs. 15.7±1.1% in JP4-039 in JP4-039-treated HCAEC) (Fig 1C and D). In addition, the amount of mito-ROS was measured using mitoSOX^TM^ red under hypoxia condition. Mito-ROS fluorescence was significantly decreased in hypoxic condition in JP4-039-treated HCAEC in comparison with the vehicle-treated HCAEC (Fig 1E).

### JP4-039 induces tube formation in HCAEC and EC sprouting in atrial tissues *ex vivo*

**Fig 2:**
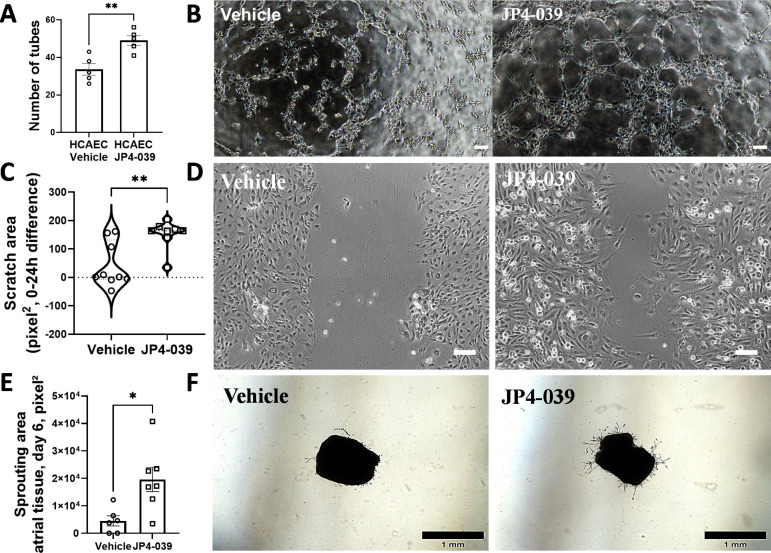
JP4-039 induced angiogenesis in HCAEC and mouse atrial tissue. (A and B) JP4-039 increased tube formation in HCAEC, 3 h after plating in a 3D BME matrix with EC medium containing 5 µM vehicle or JP4-039. n=5 wells/group. (C and D) JP4-039 improved ‘wound healing’ of HCAEC cultured under hypoxia condition. n=9 wells/group. *p<0.05, **p<0.01. (E and F) JP4-039 increased sprouting area of atrial explants. Atrial explants were harvested from NOX2-OE mice and cultured *ex vivo* in 3D Matrigel in EC media containing 5 µM of vehicle or JP4-039 for 5 days. n=7 wells/group. Results were analyzed using Shapiro-Wilk and Student’s t-test (panels A and E) or Mann-Whitney test (panel C). Scale bars are 100 µm in B, 100 µm in D, and 1 mm in F.

We next evaluated the effects of a decrease in mito-ROS on EC. To that end, we examined the effect of JP4-039 on tube formation in HCAEC *in vitro* and on EC sprouting from mouse atrial tissues *ex vivo*. HCAEC treated with JP4-039 demonstrated a 46% increase in tube formation in comparison with the vehicle (33.6±3.1 tubes in vehicle vs. 49.0±2.6 tubes in JP4-039 group) (Fig 2A and B).

In order to mimic ischemic condition *in vitro* HCAEC were incubated under hypoxic conditions in the presence or absence of JP4-039 and ‘wound healing’ (scratch assay) assay was performed. HCAEC treated with JP4-039 demonstrated significantly improved ‘wound healing’ by 86-fold under hypoxia conditions (1.9 (‐6.8;132) pixel^2^ in vehicle vs. 164 (146;177) pixel^2^ in the JP4–039 group), compared to vehicle-treated HCAEC (Fig 2C and D), suggesting increased migration of HCAEC by JP4–039 under hypoxic conditions. Interestingly, JP4–039 did not improve tube formation when HCAEC were cultured under hypoxia conditions (S2 Fig). Although the precise reason for this finding remains to be studied, it is plausible that endothelial cells in an *in vitro* three-dimensional condition may have a higher demand for oxygen during tube formation, and thus exposure to prolonged hypoxia may abrogate the beneficial effects of JP4–039 *during* tubulogenesis in matrigel *in vitro.*

Similarly, atrial tissues harvested from mice and treated with vehicle or JP4–039 in a 3D culture *ex vivo* showed a 4-fold increase in EC sprouting (outgrowth area) in comparison with the vehicle group (outgrowth area 4.5x10^3^±1.9 x10^3^ pixel^2^ in vehicle group versus 19.6x10^3^±4.4x10^3^ pixel^2^ in the JP4–039-treated group) (Fig 2E and F).

JP4-039 improves cardiac function and cardiac dilatation, and reduces infarct size in post-MI heart

**Fig 3:**
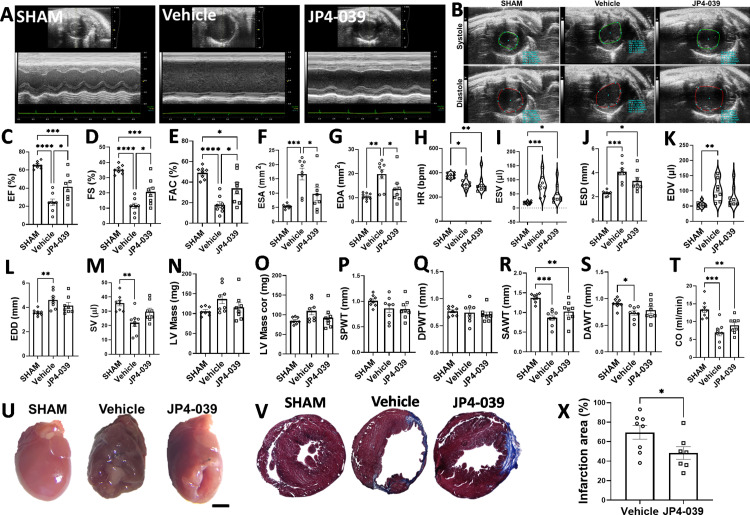
JP4-039 improves post-MI cardiac function and reduces infarction area in myocardium. Representative images of the LV (short-axis view) obtained by M-Mode echocardiography are shown in A. Panel B shows representative images of LV chamber dimensions obtained by two-dimensional echocardiography of the LV in short-axis view. The following LV function and hemodynamics parameters are shown: (C) EF, (D) FS, (E) FAC, (F) ESA, (G) EDA, (H) HR, (I) ESV, (J) ESD, (K) EDV, (L) EDD, (M) SV, (N) LV mass, (O) LV mass cor, (P) SPWT, (Q) DPWT, (R) SAWT, (S) DAWT, (T) CO. In U, example images of the hearts 28 days post-surgery are shown. Trichrome-stained heart sections (V) along with graphical result (X) show the infarcted area measured 28 days post-MI. n= 7–8 animals per group. *p<0.05, **p<0.01, *** p<0.001, **** p<0.0001. Normality of data was tested by Shapiro-Wilk. Parametric results were analyzed by Student’s t-test (panel X) or One Way ANOVA with Tukey’s post-hoc (panels C–G, J, and L–T) and represented as mean ± SEM. Non-parametric results (panels H-I, and K) were analyzed by Kruskal-Wallis followed by Dunn’s test and represented as median and minimum and maximum interquartile range. Scale bar is 1 mm. EF= ejection fraction, FS= fractional shortening, FAC= fractional area change, HR= heart rate, CO= cardiac output, ESV= end systolic volume, EDV= end diastolic volume, SV= stroke volume, ESD= end systolic diameter, EDD= end diastolic diameter, SAWT= systolic anterior wall thickness, DAWT= diastolic anterior wall thickness, SPWT= systolic posterior wall thickness, DPWT= diastolic posterior wall thickness, ESA= end systolic area, EDA= end diastolic area.

To determine the effects of mito-ROS scavenging by JP4-039 on post-MI cardiac function, we examined the left ventricle (LV) function at twenty-eight days after induction of MI (LAD surgery) or SHAM surgery in mice. To that end, animals that underwent LAD surgery were treated without (vehicle control) or with intraperitoneal injections of JP4-039 (1 mg/kg, 3 times per week for four weeks). M-mode and two-dimensional echocardiographic images acquired at short-axis view (Fig 3A and B) showed a significant decrease in ejection fraction (EF), fractional shortening (FS), and fractional area change (FAC) by 63%, 67%, and 63%, respectively, in the vehicle group compared to SHAM (Fig 3C–E). This suggested appropriate level of infarction was achieved. Animals treated with JP4–039 demonstrated significant recovery of post-MI cardiac function, as evidenced by increases in EF (by 69%), FS (by 79%) and FAC (by 89%) as compared to vehicle group (EF: vehicle=24.4±3.6 vs. JP4–039=41.3±5.2; FS: vehicle=11.5±1.7 vs. JP4–039=20.7±3.0; FAC: vehicle=18.0±3.0 vs JP4–039=33.9±5.4; Fig 3C–E). When all three groups were compared, the following measurements of post-MI cardiac functions were obtained: EF: SHAM =65.4±1.4, vehicle =24.4±3.6, JP4–039 =41.3±5.2; *p* value =0.0117 (vehicle *vs.* JP4–039); FS: SHAM =35.3±1.0, vehicle =11.5±1.7, JP4–039 =20.7±3.0; *p* value =0.0140 (vehicle *vs.* JP4–039); FAC: SHAM=49.0±2.1, vehicle=18.0±3.0, JP4–039=33.9±5.4; *p* value =0.0181 (vehicle *vs.* JP4–039; Fig 3C–E). The end systolic area (ESA) and end diastolic area (EDA) were increased by 3-fold and 1.87-fold, respectively, in the vehicle group as compared to SHAM (ESA: 16.7±2.1 in vehicle vs. 5.4±0.3 in SHAM; EDA: 19.8±2.0 in vehicle vs. 10.6±0.5 in SHAM) (Fig 3F–G), suggesting cardiac dilatation in vehicle group 28 days after MI. This cardiac dilatation was partially but significantly reversed in the animals treated with JP4-039 as shown by 42% and 31% decrease in ESA and EDA, respectively, in the JP4-039 group compared to vehicle (ESA: 9.7±2.2 in JP4–039 vs. 16.7±2.1 in vehicle; EDA: 13.6±2.1 in JP4-039 vs. 19.8±2.0 in the vehicle) (Fig 3F and G). Of note, there was no significant difference in ESA and EDA between SHAM and JP4-039 groups, suggesting effective prevention of cardiac dilatation by JP4-039 in post-MI hearts. There was no significant effect of JP4-039 on other parameters of cardiac function, such as heart rate (HR), stroke volume (SV), and LV mass, in comparison to vehicle animals (Fig 3H–T).

Next, we examined the effects of JP4–039 on the size of myocardial infarct area. Representative images of hearts harvested 28 days post-MI (Fig 3U) and subject to histological examination of heart sections stained with Masson’s Trichrome demonstrated that JP4–039 group had 31% smaller infarction area as compared to the vehicle group (69.5±7.0% in vehicle vs. 48.3±6.5 in JP4–039) (Fig 3V–X).

### JP4-039 induces coronary angiogenesis in the ischemic region of post-MI heart

**Fig 4:**
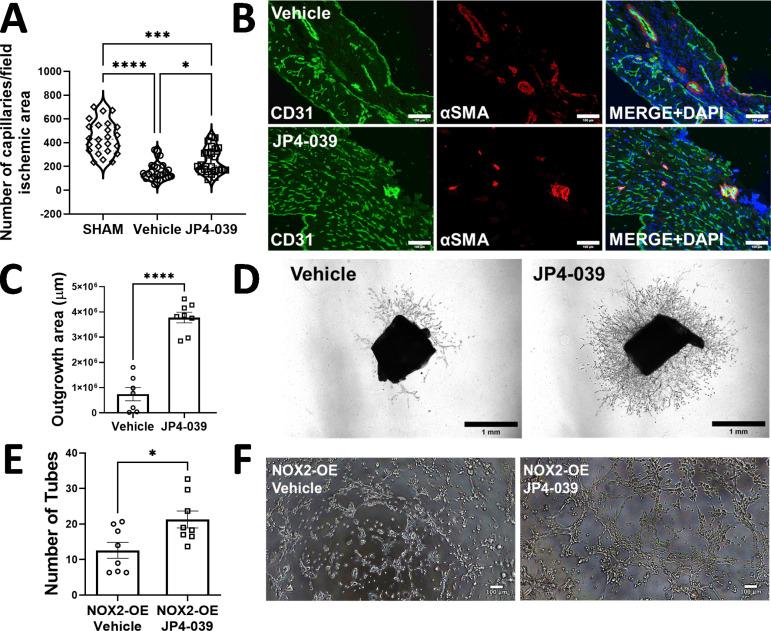
Treatment with JP4-039 induced angiogenesis *in vitro, ex vivo and in vivo.* In A and B, JP4-039 increased capillary density in the ischemic area of FVB mouse hearts 28 days after MI surgery. n= 8 animals/group. In C and D, JP4-039 increased sprouting area of aortic explants. Aortic explants were harvested from FVB mice after *in vivo* treatment with vehicle or JP4-039 for 4 weeks and cultured *ex vivo* in 3D Matrigel for 5 days. JP4-039 was used *in vivo* 1 mg/kg, injected intraperitoneally 3 times per week for 28 days after MI surgery. n= 8 animals/group. In E and F, JP4-039 increased tube formation in mouse NOX2-OE MHEC, a transgenic lineage that presents mitochondrial dysfunction and reduced EC sprouting after prolonged exposure to increased ROS; data are from 3 hours after plating MHEC in 3D matrigel with EC medium containing 5 µM of JP4-039 or vehicle. n= 8 animals/group. *p<0.05, *** p<0.001, **** p<0.0001. Normality of data was tested by Shapiro-Wilk. Non-parametric results (panel A) were analyzed by Kruskal-Wallis followed by Dunn’s test and represented as median and minimum and maximum interquartile range. Parametric results (panels C and E) were analyzed by Student’s t-test and represented as mean ± SEM. Scale bar is 100 µm in B and F, and 1 mm in D.

Our recent study demonstrated that EC-specific reduction in mitochondrial ROS in a binary conditional transgenic animal resulted in coronary angiogenesis in post-MI ischemic myocardium [21]. We therefore wanted to examine whether improvement in post-MI cardiac function by JP4–039 was associated with increased coronary angiogenesis. To that end, we examined capillary and arteriole densities in heart sections from animals that underwent LAD ligation (MI model) and were treated with or without JP4-039, using anti-CD31 and anti-smooth muscle actin (α-SMA) antibodies for immunofluorescence assays. The vehicle group demonstrated a decrease in capillary density (by 69%) in the ischemic regions of heart compared to SHAM group [SHAM= 430.0 (330.5; 543.8), vehicle= 135 (104.3; 199.3), JP4-039= 206.5 (166.8; 340.3)] (Fig 4A and B). Ischemic regions of the hearts from the JP4-039 animal group demonstrated increase in capillary density by 35% compared to the vehicle group [JP4-039= 206.5 (166.8; 340.3), vehicle= 135 (104.3; 199.3)] (Fig 4A and B), suggesting increased coronary angiogenesis by JP4-039.

We next harvested aortae from the mice that were treated with JP4-039 for 28 days and subject them to aortic sprouting assays in *ex vivo* assays as described [33]. Aortae from JP4-039-treated animals showed a 5-fold increase in EC sprouting as compared to aortae from the vehicle group (outgrowth area was 7.5×10^5^±2.6×10^5^ µm in the vehicle group versus 37.8 ×10^5^±2.1×10^5^ µm in the JP4-039 group) (Fig 4C and D). Finally, we tested the effects of JP4-039 in MHEC. To that end, we isolated MHEC from NOX2 (NADPH Oxidase 2 or gp91^phox^) overexpression (OE) mice that have EC-specific increase in ROS. NOX2-OE was obtained using a binary conditional transgenic mice as described earlier [6]. The EC-specific increase in ROS was shown in MHEC from NOX2-OE mice in a previous study [6]. MHEC from NOX2-OE animals show impaired angiogenesis (tube formation) *in vitro* (Fig 4E and F). JP4-039 increased tube formation (by 69%) in NOX2-OE MHEC (12.6±2.2 tubes in the vehicle-treated group versus 21.3±2.4 tubes in the JP4–039-treated group (Fig 4E and F), suggesting that reduced mito-ROS significantly improved angiogenic potential of MHEC with oxidative stress.

JP4-039 reduced AMPKα phosphorylation in cardiac tissue, and increased the expression of mitochondrial complexes I and V, and ATP synthesis in EC

**Fig 5:**
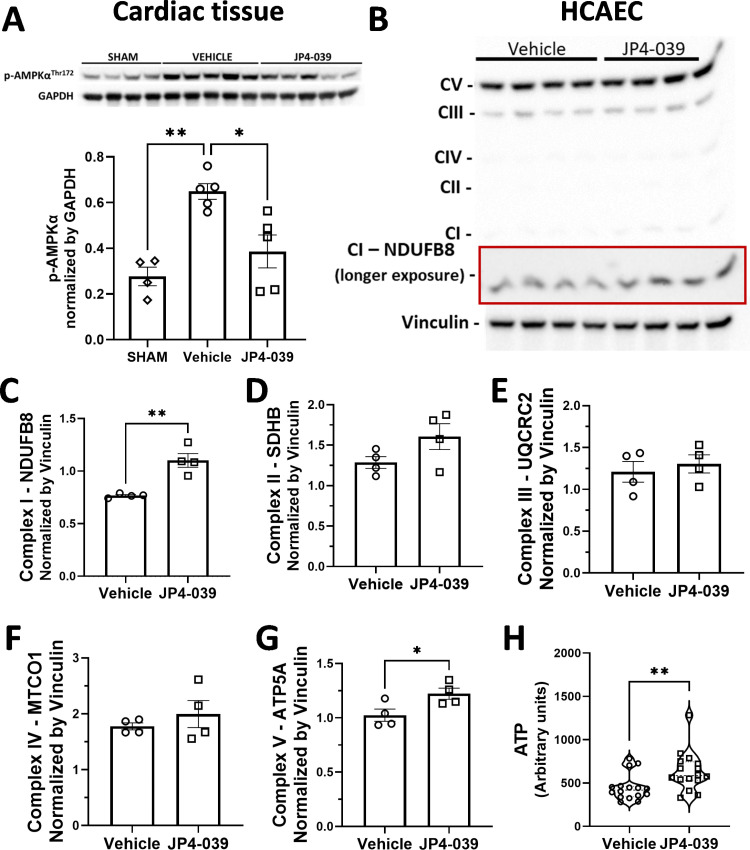
JP4-039 reduces AMPK **α** **phosphorylation, increases the expression of mitochondrial complexes I and V, and ATP content in EC.** (A) Representative western blot bands and quantitative results of p-AMPKα measured in heart homogenates from the infarcted mice treated with or without JP4-039, obtained 28 days post-MI, are shown. n= 4 to 5 heart homogenates/group (each band is the result from one individual heart). (B) Representative image of OXPHOS western blot measured in HCAEC lysates after a three-hour treatment with vehicle or 5 µM JP4-039 under starvation condition. (C–G) Quantitative analyses of mitochondrial complexes I (C), **II** (D), **III** (E), **IV** (F), and V (G). n= 4 plates/group. (H) ATP content was measured by ATP luminescence assay in HCAEC after three-hour treatment with vehicle or 5 µM JP4-039. n= 16 wells/group. *p<0.05, **p<0.01. Normality of data was tested by Shapiro-Wilk. Parametric results were analyzed by One Way ANOVA with Tukey’s post-hoc (panel A) or by Student’s t-test (panels C–G) and represented as mean ± SEM. Nonparametric results were analyzed by Mann-Whitney test (panel H) and represented as median and minimum and maximum interquartile range.

To examine the effects of JP4-039 on signaling pathways, western blots were performed using cardiac tissue (non-ischemic region). Cardiac tissue of the post-MI mice that were treated with vehicle demonstrated 2.3-fold increase in phosphorylation of AMPKα at the Threonine 172 residue when compared to SHAM group (0.28±0.04 in SHAM group versus 0.65±0.03 in vehicle) (Fig 5A). Post-MI cardiac tissue lysates from the JP4-039 group showed a decrease in p-AMPKα as compared to the vehicle group (0.65±0.03 in vehicle versus 0.39±0.07 in JP4-039) (Fig 5A). There was no significant difference in AMPKα phosphorylation between SHAM and JP4-039 groups (Fig 5A).

Since reduced phosphorylation of AMPKα by JP4-039 may reflect an altered AMP:ATP ratio and since JP4-039 is a mitochondria-specific antioxidant, we next determined the expression levels of mitochondrial complexes involved in electron chain transport (ETC) and oxidative phosphorylation (OXPHOS) using HCAEC treated with or without JP4-039 (Fig 5B). Using Western blots analyses, HCAEC treated with JP4-039 demonstrated a 44% increase in mitochondrial complex I expression (0.77±0.01 in vehicle versus 1.10±0.07 in JP4-039) (Fig 5C). Expression of mitochondrial complexes II, III, and IV did not change with JP4-039 treatment (Fig 5D–F). However, the expression of the mitochondrial complex V (ATP synthase) was 19% higher in JP4-039 than in the vehicle-treated HCAEC (1.02±0.06 in vehicle versus 1.22±0.05 in JP4-039, Fig 5G). We next decided to determine the ATP content in HCAEC. JP4-039-treated HCAEC showed 40% increase in ATP luminescence as compared to vehicle-treated HCAEC (ATP luminescence was 419.5 (342.6; 463.8) in vehicle versus 585.0 (533.7; 744.1) in the JP4-039 group), suggesting a significant increase in ATP content of EC that were treated with JP4-039 (Fig 5H).

## Discussion

A growing body of research has found mitochondrial dysfunction at the center stage of pathophysiology of CVDs including MI [4,34,35]. In the current study, we tested the systemic administration of a mitochondria-specific antioxidant nitroxide compound, JP4-039, to examine the effects of mito-ROS reduction on coronary angiogenesis in post-MI ischemic myocardium and on cardiac function recovery. We demonstrate that JP4-039-mediated reduction in mito-ROS reduces infarct size, improves coronary vessel density in ischemic myocardium and results in significant recovery of cardiac function in post-MI animals. JP4-039 also induces angiogenesis, and increases expression of mitochondrial complexes I and V, and ATP levels in coronary ECs, suggesting a novel mechanism may be in play to provide resilience to coronary endothelium and ischemic myocardium resulting from the decreased mito-ROS in post-MI heart.

Our recent study demonstrated that a vascular endothelium-specific genetic reduction in mitochondrial ROS resulted in improved coronary angiogenesis in ischemic myocardium *in vivo* [21]. In that study, we also showed that decreased mito-ROS resulted in increased mitochondrial complexes expression, mitochondrial supercomplex formation, and oxidative phosphorylation in coronary ECs [21]. In the current study, we observed similar effects on coronary angiogenesis and cardiac function recovery in post-MI hearts by systemic administration of the mitochondria-specific nitroxide compound JP4–039 *in vivo*. We also report here that reduction in mito-ROS in human coronary EC (HCAEC) by JP4–039 resulted in increase in the expression of mitochondrial complexes I and V with a significant increase in ATP synthesis *in vitro*. Corroborating with these findings, JP4-039 was shown to improve basal respiration and reserve capacity of human skin fibroblasts with very long-chain acyl-CoA dehydrogenase (VLCAD) or complex I deficiency [36,37].Together, our findings suggest that reduction in mito-ROS in vascular endothelium may play a significant role in JP4-039-mediated improvement in coronary angiogenesis and recovery of post-MI cardiac function *in vivo*. However, further studies are required to elucidate the precise roles of JP4-039-mediated reduction in mito-ROS specifically in vascular endothelium and in cardiomyocytes in ischemic heart *in vivo.* In order to determine specific contributions of reduction in mito-ROS by JP4-039 in endothelial cells versus cardiomyocytes (and other cell types) to induce coronary angiogenesis and recovery of cardiac function, spatial transcriptomic studies involving post-MI hearts are being carried out in our lab, along with siRNA studies *in vitro*. These ongoing studies will also help elucidate the molecular mechanisms including metabolic shift from glycolysis to oxidative phosphorylation (OxPhos) that may play major roles in providing resilience to coronary EC during myocardial ischemia by JP4-039.

**Fig 6:**
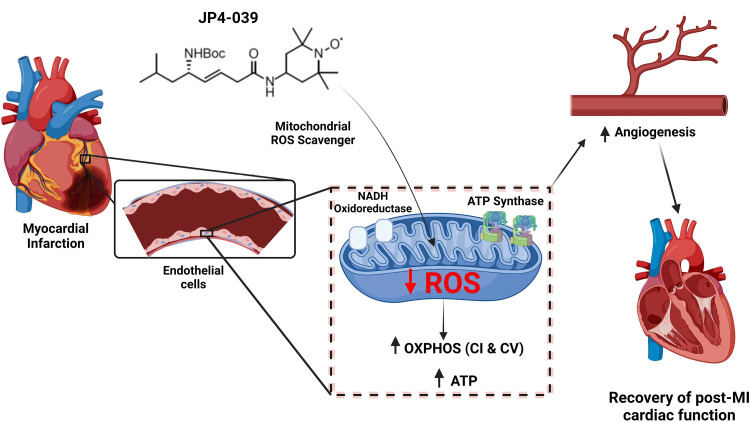
Interpretation of findings of the present study. In acute MI, the exacerbation of Mito-ROS leads to mitochondrial dysfunction and a decrease in ATP production, leading to an increase in AMP/ATP ratio, which results in phosphorylation of AMPKα at Threonine 172. Over time, the unmet need for ATP results in impaired angiogenesis and loss of capillaries in the heart, affecting blood supply and myocardial healing. This leads to cardiac maladaptation, resulting in impaired cardiac function associated with larger infarcted tissue area after acute MI. When JP4-039 is introduced after acute MI, its mitochondria-targeted ROS and electron scavenging activities result in a reduction of Mito-ROS and increased expression of mitochondrial complexes I and V. Thus, treatment with JP4-039 results in increased ATP supply and reduced phosphorylation of AMPKα at Threonine 172 in comparison to untreated mice after MI, suggesting an improvement of the AMP/ATP balance. The increase in ATP supply promoted by JP4-039 may contribute to angiogenesis associated with an improved cardiac function and reduction of infarct size after an acute MI. Created with BioRender.com.

Other groups have also suggested that increasing mitochondrial OXPHOS and thereby improving ATP synthesis in cardiomyocytes would be beneficial in CVD settings such as MI and heart failure [4]. A considerable effort has been spent in the search for a treatment that can increase ATP supply and improve cardiac function [38–40]. Mitochondria-targeted antioxidants and SOD mimetics have been studied in preclinical models of CVD with promising results [11,41,42]. However, this line of research so far has mostly targeted cardiomyocyte and their recovery during ischemia. Consequently, the role of the coronary endothelium, comprised of endothelial cells that modulate fundamental functions to preserve myocardial oxygen and nutrient supply, has been under-investigated [43]. Our prior research has shown that overexpression of MnSOD (a mitochondria antioxidant enzyme) in EC-specific manner induced increases in assembly of supercomplexes, mitochondrial respiration, and oxidative phosphorylation in coronary ECs [21]. These findings were accompanied by induction of coronary angiogenesis and cardiac function recovery after MI [21]. In the present study, the effect of the mitochondria-targeted ROS scavenger JP4-039 was investigated for cardiac function recovery in post-MI animals. Interestingly, this study shows for the first time that the mitochondrial antioxidant JP4-039, decreases ROS and increases the expression of mitochondrial complexes I and V, resulting in increased ATP content and migration as demonstrated by wound healing in HCAEC. Our finding that JP4-039 decreases mito-ROS in HCAEC reinforces a previous report by us in which JP4-039, directly or released from an electrospun nanofibrous scaffold, was found to decrease mito-ROS in HCAEC [18]. These findings, summarized in Fig 6, emphasize the importance of the endothelium in the improvement of cardiac function and further support the potential of mitochondria-targeted drugs to ameliorate energy supply in cardiovascular disease settings.

Although the results shown here demonstrate that reduction in mito-ROS increases expression of mitochondrial complexes and synthesis of ATP, the precise mechanism by which reduced mito-ROS increases the OxPhos complexes is not yet known. Our ongoing studies using spatial transcriptomics in post-MI myocardium treated with vehicle or JP4-039 may address this query in the future.

We have previously reported that JP4-039 induces angiogenesis in HCAEC [18], and now demonstrated for the first time that this effect is also present in mouse atrial tissue *ex vivo* and that JP4-039 improves vessel density in mouse ischemic heart tissue *in vivo.* Similarly, another research group demonstrated that SS-31 rescued cerebrovascular endothelial function in aged mice [44]. Mito-TEMPO has also been shown to improve coronary endothelial function in a cardioplegic ischemia-reperfusion-induced injury model [45]. Interestingly, while JP4–039 may not improve tube formation in HCAECs under hypoxia conditions *in vitro*, it is still capable of enhancing cell migration, an event that is major characteristic feature of EC angiogenesis [46]. Additionally, EC migration has been shown to contribute to re-endothelization in an *in vitro* injury model at a 2 mm injury scale after stent placement [47]. Notably, mitochondria targeted antioxidants can act not only by inducing angiogenesis but have also been reported to be improving coronary endothelial function in CVD [45]. The changes in mito-ROS and coronary angiogenesis found in the present study were associated with a significantly smaller infarction area and a substantial improvement in cardiac function in post-MI mice treated with JP4–039. Corroborating our data, an improvement in parameters of cardiac function has been demonstrated in an *ex vivo* ischemia/reperfusion (I/R) model after treatment with the highly targeted mito-ROS scavenger XJB-5–131 [11,48]. Similarly, a reduction in frequency and severity of arrhythmias, along with reduced infarction area was previously described in rats pre-treated with mitochondrial-targeted peptides SS-31 and SS-20 prior to a non-survival I/R model [13]. Studies using mito-TEMPO have also demonstrated that mito-ROS scavenging offers protection from arrhythmogenesis [49,50]. Our current report, using a non-reperfused MI (permanent LAD ligation) model, reinforces the findings of the previous studies that used I/R and other models and demonstrates a future potential translational application for patients with myocardial ischemia or an MI event, regardless of timely reperfusion therapy. Furthermore, the fact that JP4-039 was able to increase the expression of mitochondrial complexes I and V, and the ATP content in endothelial cells, along with the finding that the myocardial tissue of infarcted animals treated with JP4-039 presented a decrease in the phosphorylation of AMPKα, suggests that JP4-039 may be able to recover, at least in part, the impairment in AMP/ATP ratio and decrease in energy supply that follows an MI event. Phosphorylation of AMPKα at the threonine 172 site is required for AMPKα activation, which often occurs during events of stress, such as hypoxia and ischemia, which deplete ATP content resulting in an increase in AMP:ATP ratio [51–53]. Once activated, AMPKα modulates energy metabolism by activating pathways that generate ATP and downregulating pathways associated with ATP consumption [52,53]. As reported by Choi and collaborators, AMPKα is activated by H2O2 in a concentration-dependent matter that is tightly associated with the AMP:ATP ratio [54]. Since JP4-039 is a mito-ROS scavenger and was able to reduce ROS and improve ATP content, it is plausible that JP4-039 offered protection by shifting the AMP:ATP ratio in favor of ATP, thus reducing the necessity for activation of AMPKα.

On a drug safety aspect, while there have been no studies investigating potential off-target effects of JP4-039, long-term studies have been conducted in animal models. One study conducted ten independent experiments over a five-month period to evaluate the reproducibility of intramuscular (IM) delivery of JP4-039 in different formulations for radiation mitigation in mice [55]. JP4-039 consistently demonstrated improved survival rates after irradiation when delivered IM in cyclodextrin or Miglyol-812-N formulations across multiple experiments [55]. Another long-term study examined the effects of JP4-039 on mouse survival and intestinal recovery after total body irradiation (TBI). Mice were monitored for up to 35 days after TBI and found that a single administration of JP4-039 24 hours after irradiation significantly improved survival rates from 10–25% to 75% by Day 35 [56]. A third study evaluated the effectiveness of JP4-039 and its analogs as TBI mitigators in mice and found that treatment with JP4-039 or its analogs improved mouse survival 35 days after irradiation [17].Our findings that reduction in mito-ROS improves angiogenic potential of coronary endothelium in ischemic myocardium *in vivo*, in atrial tissue ex vivo and in HCAEC *in vitro* are in apparent discrepancy with previous studies showing that an increase in mito-ROS by adding exogenous H_2_O_2_ or Nox4 stimulation induced proliferation and migration in the human umbilical vein (HUVEC) *in vitro* [57]. This apparent discrepancy with our current study may be addressed by the findings of a recent study by Carmeliet’s group which showed that heterogeneity of EC transcriptional profiles includes differences in VEGF signaling, ROS metabolism and EC proliferation across ECs from different tissues [58]. These tissue-specific signatures and inter-tissue heterogeneity of ECs based on the tissue of origin may explain difference in responses to sub-cellular ROS signaling in HUVEC versus the current study that used coronary ECs from human (HCAEC) and mice (MHEC).

Although the current study presents interesting findings, there are several limitations which need to be further studied in the future. One limitation of this study is the absence of comorbidities (hypertension, metabolic syndrome, diabetes, etc) in our animal model with MI. These comorbidities are common in the clinical setting of cardiovascular disease. A model of type-II diabetes or metabolic syndrome, for instance, will provide translational data in the ischemic setting. Our ongoing studies will address these comorbidities in our future animal models to enhance the translational potential of the study. Another mitochondria-targeted antioxidant, MitoQ, has been demonstrated to improve cardiac function in a type II diabetic rat model of I/R [59]. Furthermore, a randomized clinical trial has demonstrated that hypertensive individuals subjected to exercise training or exercise training in combination with oral administration of MitoQ presented improvements in left ventricular mass and end-systolic and diastolic diameters [60]. Another important limitation is that although the current study showed significant beneficial effects of the mitochondria-targeted antioxidant JP4-039 on ischemic myocardium, our current data do not provide information on specific cell types’ involvement in this outcome. As such, the effects of JP4-039 on cardiac EC, cardiomyocytes, cardiac fibroblasts and other cell types, and their relative contributions to an improved post-MI recovery of cardiac function remain to be determined. However, prior studies have reported a reduction in apoptosis and improved integrity of the mitochondrial cristae in H9C2 cells treated with MitoQ [59,61]. Interestingly, using cardiac fibroblasts, a study by Janbandhu and collaborators has shown that MitoTEMPO reduced the proliferation in cardiac fibroblasts four weeks post-MI [62]. Finally, the current study used a permanent ligation of LAD artery in mice, which may not fully reflect clinical scenarios where there is reperfusion therapy. As discussed earlier, other mitochondria-targeted antioxidants, such as XJB-5–131, SS-31, and SS-20, have been studied in preclinical *ex vivo* and I/R models [11,13,48]. It would be interesting to examine the potential *in vivo* benefits of JP4-039 using I/R model in future. Taken together, the results presented in the current study suggest that nitroxide compound-mediated reduction in mito-ROS may help develop therapeutic modalities for myocardial ischemia.

## Supporting information

S1 Fig Experimental designs including in vitro, ex vivo, and in vivo studies. Upper panel, *In vitro* study was conducted using HCAEC and NOX2-OE MHEC (1). ECs were treated for 1.5 or 3 hours with vehicle or JP4–039, followed by assays of (2) mitochondrial redox status (mito-roGFP), angiogenesis (tube formation), and Western blot analysis. Lower panel, *in vivo* studies using LAD ligation (MI) in mice followed by intraperitoneal injections of vehicle or JP4–039 for four weeks. Echocardiography for cardiac function, histology for infarct size and coronary vascular density in ischemic myocardium, and *ex vivo* aortic and atrial explants for EC sprouting assays were employed. Created with BioRender.com.
(TIFF)

S2 Fig JP4–039 did not improve tube formation under hypoxia condition. HCAECs were seeded and incubated under hypoxia, with EC medium containing 5 µM of JP4–039 or vehicle. After 24 hours, the number of tubes was quantified in A, with representative images shown in B. Results were analyzed by Shapiro-Wilk and Student’s t-test.
(TIFF)

S1 Table Information on materials and supplies. (PDF)

S1 File Raw western blot images. (PDF)
