# Supplementary figures and images for: Mitochondria-targeted ROS scavenger JP4-039 improves cardiac function in a post-myocardial infarction animal model and induces angiogenesis in vitro
Source: PLoS One. 2025 Apr 24;20(4):e0320703. doi: 10.1371/journal.pone.0320703 (PMC12021227; doi:10.1371/journal.pone.0320703)

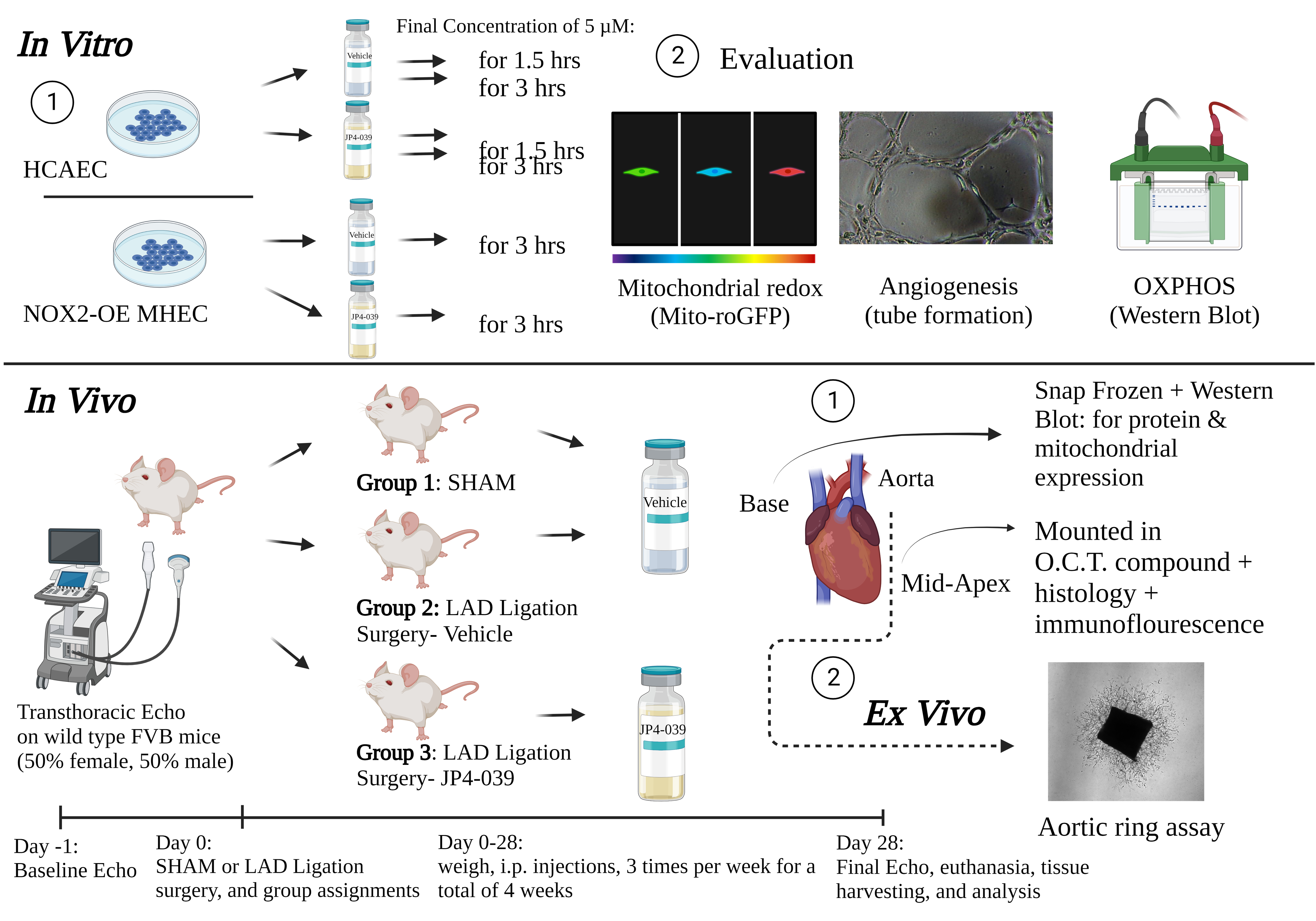

Supplement: S1 Fig — Upper panel, In vitro study was conducted using HCAEC and NOX2-OE MHEC (1). ECs were treated for 1.5 or 3 hours with vehicle or JP4–039, followed by assays of (2) mitochondrial redox status (mito-roGFP), angiogenesis (tube formation), and Western blot analysis. Lower panel, in vivo studies using LAD ligation (MI) in mice followed by intraperitoneal injections of vehicle or JP4–039 for four weeks. Echocardiography for cardiac function, histology for infarct size and coronary vascular density in ischemic myocardium, and ex vivo aortic and atrial explants for EC sprouting assays were employed. Created with BioRender.com. (TIFF) [file pone.0320703.s001.tiff]

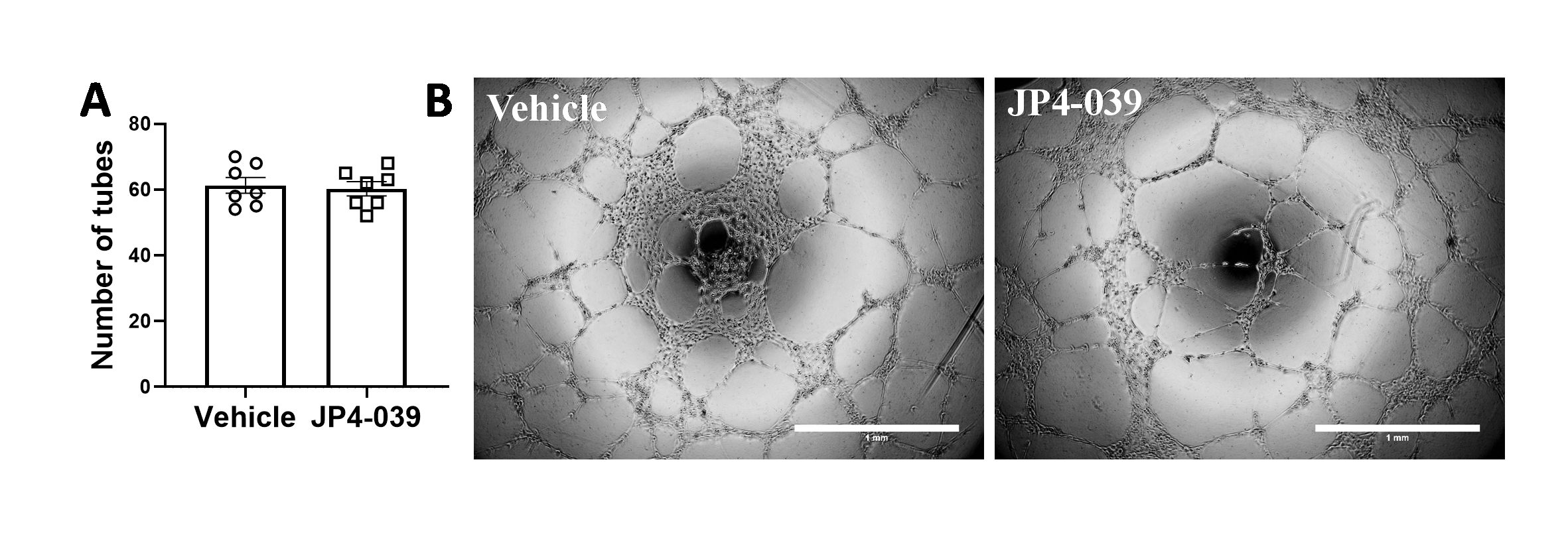

Supplement: S2 Fig — HCAECs were seeded and incubated under hypoxia, with EC medium containing 5 µM of JP4–039 or vehicle. After 24 hours, the number of tubes was quantified in A, with representative images shown in B. Results were analyzed by Shapiro-Wilk and Student’s t-test. (TIFF) [file pone.0320703.s002.tiff]
