# Supplementary material for: Mitochondria-targeted ROS scavenger JP4-039 improves cardiac function in a post-myocardial infarction animal model and induces angiogenesis in vitro
Source: PLoS One. 2025 Apr 24;20(4):e0320703. doi: 10.1371/journal.pone.0320703 (PMC12021227; doi:10.1371/journal.pone.0320703)
Supplement: S1 Table — (PDF) [file pone.0320703.s003.pdf]

S1 Table

Antibodies				
**Product**	**Source**	**Catalog #**	**Working concentration**	**ID/ URL**
Purified Rat Anti-Mouse CD31 Clone MEC 13.3 (RUO)	BD Biosciences	Catalog# 550274	1:100	Entrez Gene ID: 18613
α-Smooth Muscle Actin (D4K9N) XP® Rabbit mAb	Cell signaling	Catalog# 19245	1:100	UniProt ID: P62736<br>Entrez-Gene Id: 59
Anti-rat IgG (H+L), (Alexa Fluor® 488 Conjugate)	Cell signaling	Catalog# 4416	1:200	https://www.cellsignal.com/products/secondary-antibodies/anti-rat-igg-h-l-alexa-fluor-488-conjugate/4416
Anti-rabbit IgG (H+L), F(ab')2 Fragment (Alexa Fluor® 594 Conjugate)	Cell signaling	Catalog# 8889	1:200	https://www.cellsignal.com/products/secondary-antibodies/anti-rabbit-igg-h-l-f-ab-2-fragment-alexa-fluor-594-conjugate/8889
Phospho-AMPKα (Thr172) (40H9) Rabbit mAb	Cell signaling	Catalog# 2535	1:1000	UniProt ID: Q13131, P54646<br>Entrez-Gene Id: 5562, 5563
GAPDH (D4C6R) Mouse mAb	Cell signaling	Catalog# 97166	1:1000	UniProt ID: P04406<br>Entrez-Gene Id: 2597
Total OXPHOS rodent antibody cocktail	Abcam	Catalog# ab110413	1:1000	https://www.abcam.com/total-oxphos-rodent-wb-antibody-cocktail-ab110413.html
Vinculin Polyclonal Antibody	Thermo Fisher	Catalog# PA5-29688	1:1000	UniProt ID: (Human) P18206, (Mouse) Q64727, (Rat) P85972<br>Entrez Gene ID: (Human) 7414, (Mouse) 22330, (Rat) 305679
Anti-rabbit IgG, HRP-linked Antibody	Cell signaling	Catalog# 7074	1:5000	https://www.cellsignal.com/products/secondary-antibodies/anti-rabbit-igg-hrp-linked-antibody/7074?_=1672867381198&Ntt=ANTI%20RABB&tahead=true
Anti-mouse IgG, HRP-linked Antibody	Cell signaling	Catalog# 7076	1:5000	https://www.cellsignal.com/products/secondary-antibodies/anti-mouse-igg-hrp-linked-antibody/7076?_=1672867400152&Ntt=ANTI%20MOUSE&tahead=true
**Other Reagents**				

**Description**	**Source**	**Catalog #/ URL**
JP4-039	Dr. Peter Wipf's lab	N/A
2,2,2-Trifluoroethanol	TCI	Catalog # T0435
Sunflower seed oil from Helianthus annuus	SIGMA	SKU# S5007-250ML
EGMTM -2 MV Microvascular Endothelial Cell Growth Medium-2 BulletKit	Lonza	Catalog# CC-3202
Dimethyl Sulfoxide (DMSO)	Fisher Scientific	Catalog# BP231-100
MitoSox Red	Thermo Fisher Scientific	Catalog# M36008
Plasmid: Matrix-roGFP (Mito-roGFP adenovirus)	Addgene	Catalog# 49437
Cultrex Reduced Growth Factor Basement Membrane Extract, PathClear	R&D Systems	Catalog# 3433-005-01
µ-Slide 15 Well 3D	Ibidi	Catalog# 81506
Scigen Tissue-Plus™ O.C.T. Compound	Fisher Scientific	Catalog# 23-730-571
1X DPBS	Thermo Fisher Scientific	Catalog# 14190144
µ-dish35mm, high glass bottom	Ibidi	Catalog# 81158
ATPlite 1step luminescence assay system	Perkin Elmer	Catalog# 6016736
Masson's Trichrome stain kit	Polysciences	Catalog# 25088-100
RIPA buffer	Sigma	Catalog# R0278-50ML
protease inhibitor cocktail	SIGMA	Catalog# P8340
phosphatase inhibitor cocktail 1	SIGMA	Catalog# P5726
phosphatase inhibitor cocktail 2	SIGMA	Catalog# P0044
Sodium Fluoride (Powder/Certified ACS), Fisher Chemical™	Fisher Scientific	Catalog# S299-100
Sodium Vanadate (Laboratory), Fisher Chemical™	Fisher Scientific	Catalog# S454-50
Micro BCA™ Protein Assay Kit	Thermo Fisher Scientific	Catalog# 23235
Dithiothreitol (DTT)	Bio-Rad	Catalog# 1610611
3% Hydrogen Peroxide (H2O2)	Target	Catalog# A-15115908
0.025% Trypsin/EDTA solution (for HCAEC)	Lonza	Catalog# CC-5012

Trypsin Neutralizing solution (TNS, for HCAEC)	Lonza	Catalog# CC-5002
MitoSOX™ Mitochondrial Superoxide Indicators, for live-cell imaging	Thermo Fisher	Catalog# M36008
Corning® 96 Well Black Polystyrene Microplate	Millipore Sigma	Catalog# CLS3603-48EA
CytoOne Multiple Well Plates, TC Treated, 12-well	USA Scientific	Catalog# CC7682-7512

**Cultured Cells**			
**Cell type**	**Source**	**Sex (F, M, or unknown)**	**Catalog #/ URL**
Tet-Nox2:VE-Cad-Tta MHEC (NOX2-OE MHEC)	*In house* (Dr. Abid's lab).	M/F	N/A
HCAEC	ATCC	F	PCS-100-020 / https://www.atcc.org/products/pcs-100-020
HCAEC	ATCC	M	PCS-100-020 / https://www.atcc.org/products/pcs-100-020
HCAEC	Lonza	F	Catalog #: CC-2585

**Animals (in vivo study)**				
**Species**	**Source**	**Background Strain**	**Sex**	**Catalog # / URL**
Mouse (mus musculus)	Charles River Laboratories	FVB/NCrl 207	M/F	Strain code 207/ https://www.criver.com/products-services/find-model/fvb-mouse?region=3611
