# Supplementary material for: Mitochondria-targeted ROS scavenger JP4-039 improves cardiac function in a post-myocardial infarction animal model and induces angiogenesis in vitro
Source: PLoS One. 2025 Apr 24;20(4):e0320703. doi: 10.1371/journal.pone.0320703 (PMC12021227; doi:10.1371/journal.pone.0320703)
Supplement: S1 File — (PDF) [file pone.0320703.s004.pdf]

Original images of p-AMPKα$^{(Thr172)}$ used in Figure 5A.

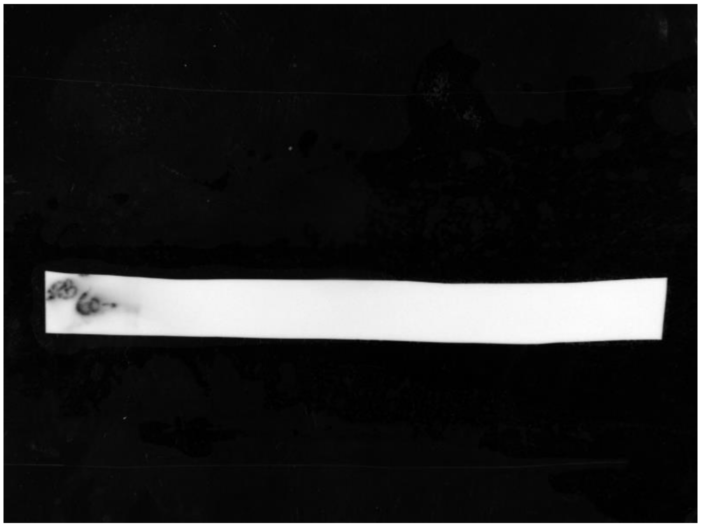

A – Original image showing membrane and molecular weight information.

Acquisition Information:

Exposure time: 0.093 seconds

Mode: Auto – Intense bands

Application: Epi

Excitation source: White epi illumination

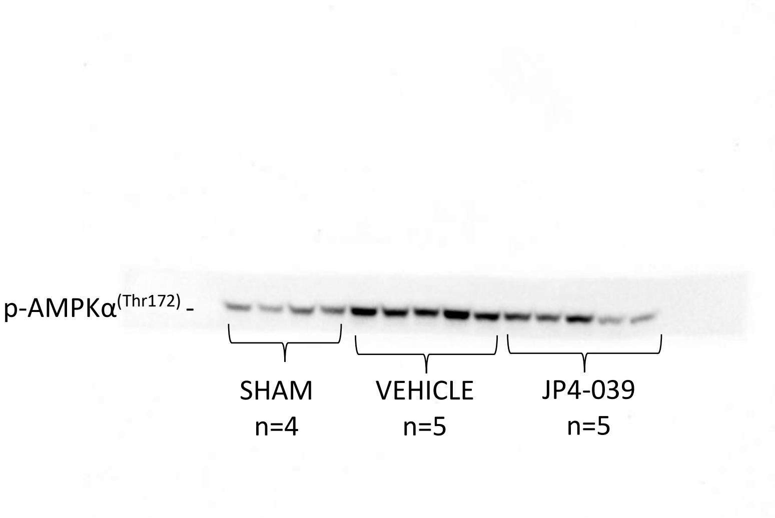

B – Original image showing p-AMPKα$^{(Thr172)}$ bands. **This is the image shown in Figure 5A.**

Acquisition Information:

Exposure time: 6.748 seconds

Mode: Auto – Intense bands

Application: Chemi Hi Sensitivity

Excitation source: No illumination

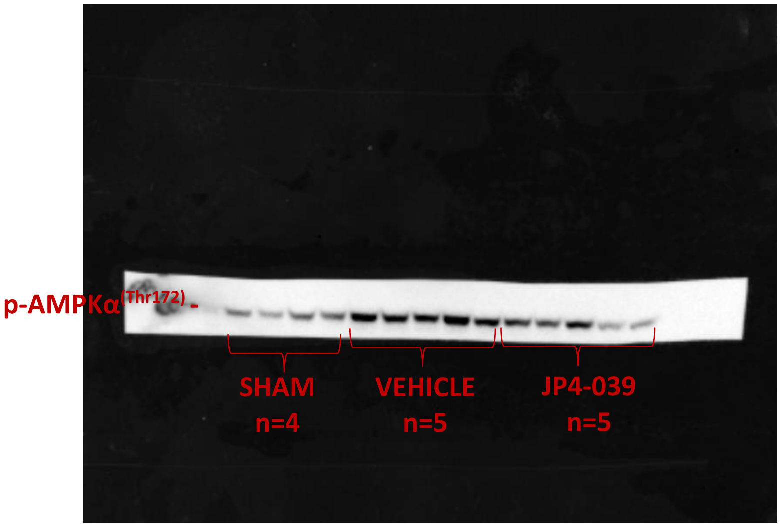

C – Merged image showing overlap of p-AMPKα$^{(Thr172)}$ bands and molecular weight marker.

Original images of GAPDH used in Figure 5A.

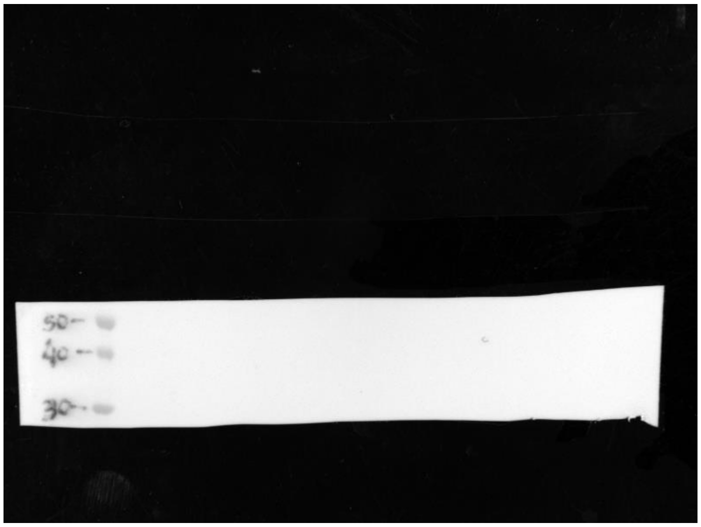

A – Original image showing membrane and molecular weight information.

Acquisition Information:

Exposure time: 0.090 seconds

Mode: Auto – Intense bands

Application: Epi

Excitation source: White epi illumination

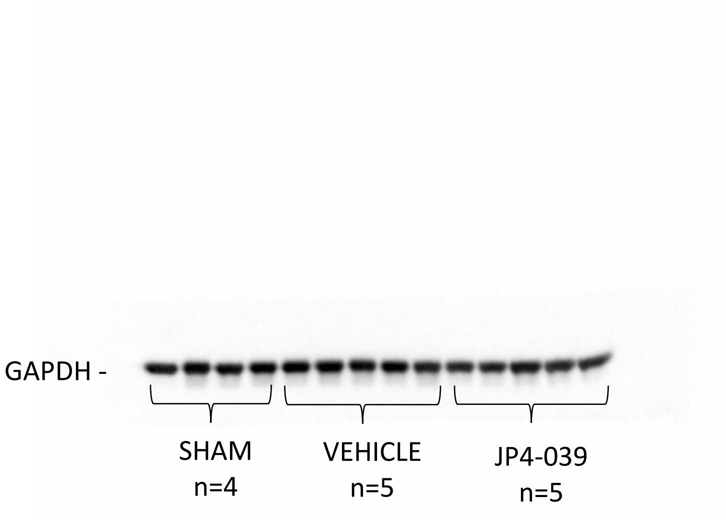

B – Original image showing GAPDH bands. **This is the image shown in Figure 5A.**

Acquisition Information:

Exposure time: 0.697 seconds

Mode: Auto – Intense bands

Application: Chemi Hi Sensitivity

Excitation source: No illumination

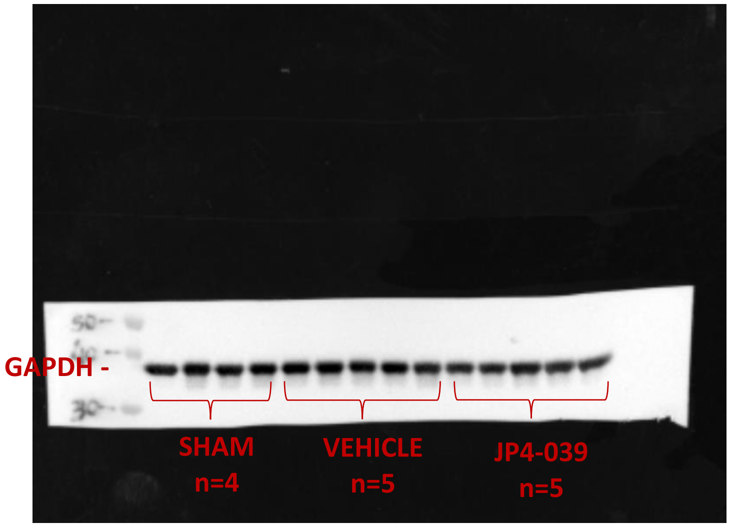

C – Merged image showing overlap of GAPDH bands and molecular weight marker.

Original images of OXPHOS antibody cocktail bands used in Figure 5B.

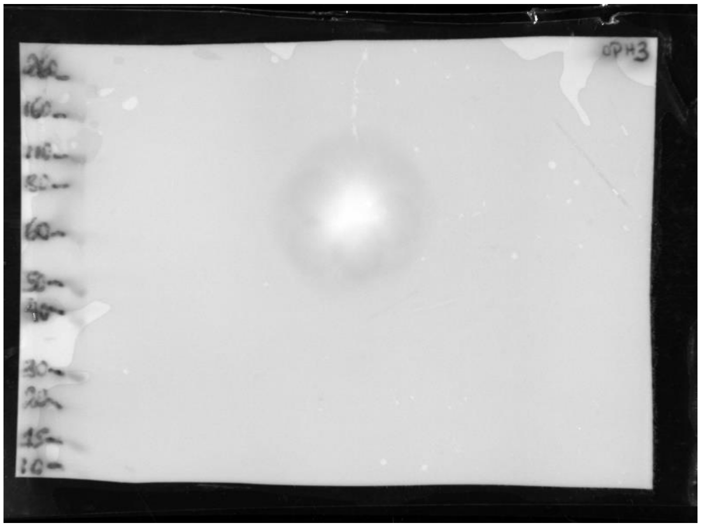

A – Original image showing membrane and molecular weight information.

Acquisition Information:

Exposure time: 0.133 seconds

Mode: Auto – Intense bands

Application: Epi

Excitation source: White epi illumination

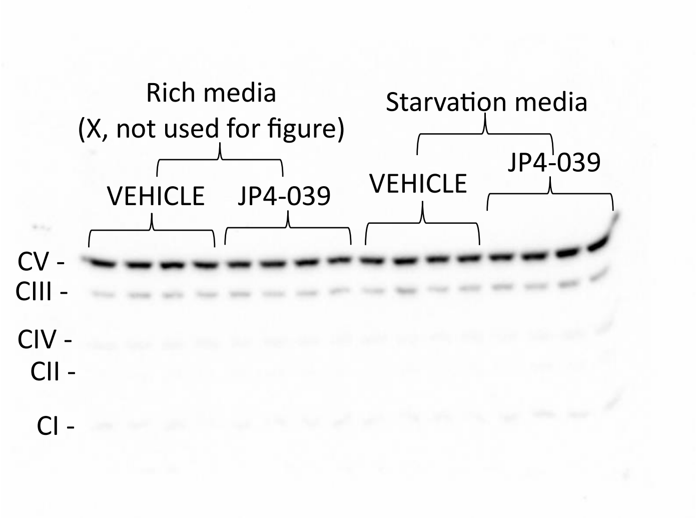

B – Original image showing Total OXPHOS bands. **This is the image shown in Figure 5B.** Bands marked with an X (columns 1-8) were not used for the figure.

Acquisition Information:

Exposure time: 3.576 seconds

Mode: Auto – Intense bands

Application: Chemi Hi Sensitivity

Excitation source: No illumination

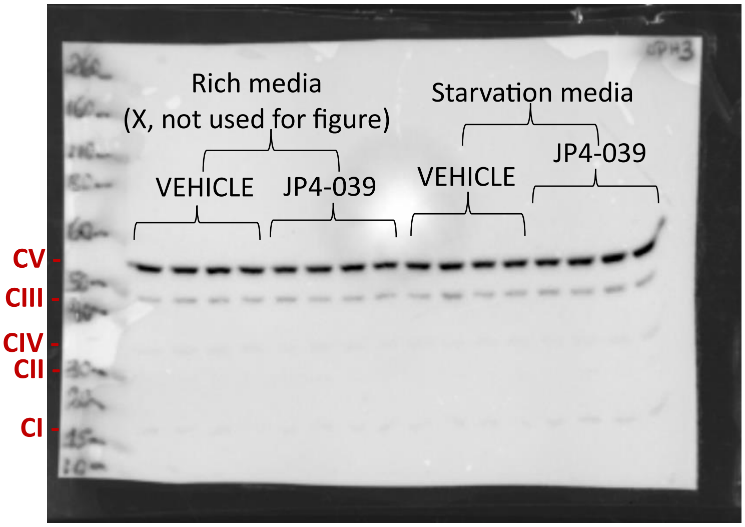

C – Merged image showing overlap of Total OXPHOS bands and molecular weight marker. Bands marked with an X were not used for the figure.

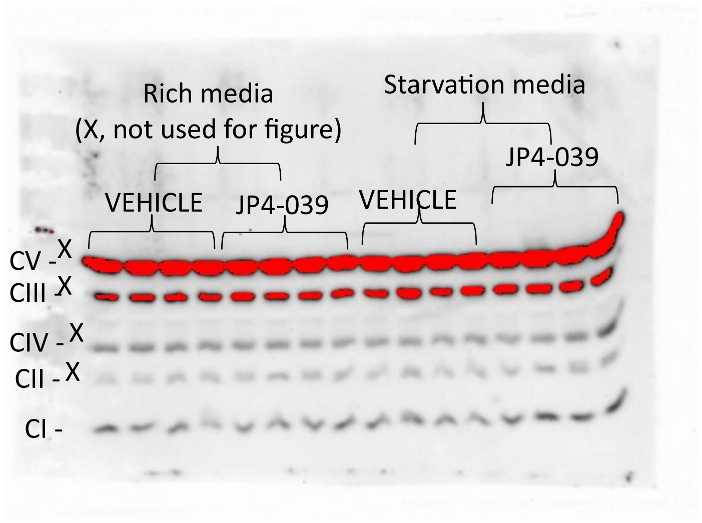

D – Original image showing Total OXPHOS bands at longer exposure. **This is the image shown in Figure 5B for CI – NDUFB8 (longer exposure).** Note that red color marks overexposed bands. Bands marked with an X were not used for the figure.

Acquisition Information:

Exposure time: 50 seconds

Mode: Manual

Application: Chemi Hi Sensitivity

Excitation source: No illumination

Original images of Vinculin bands used in Figure 5B

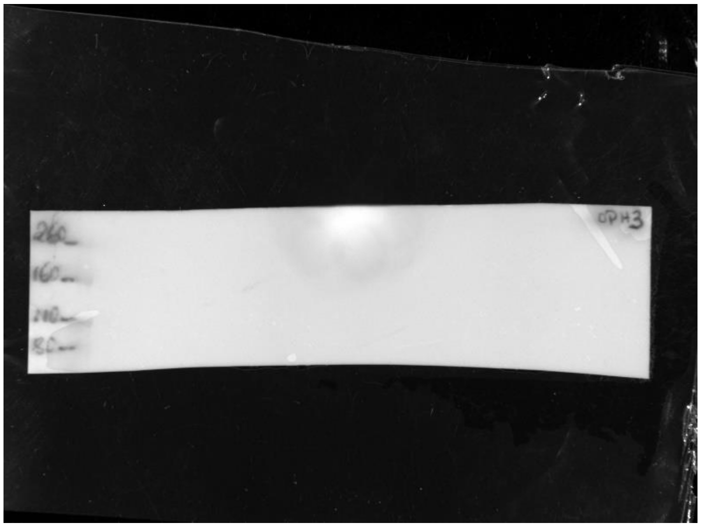

A – Original image showing membrane and molecular weight information.

Acquisition Information:

Exposure time: 0.130 seconds

Mode: Auto – Intense bands

Application: Epi

Excitation source: White epi illumination

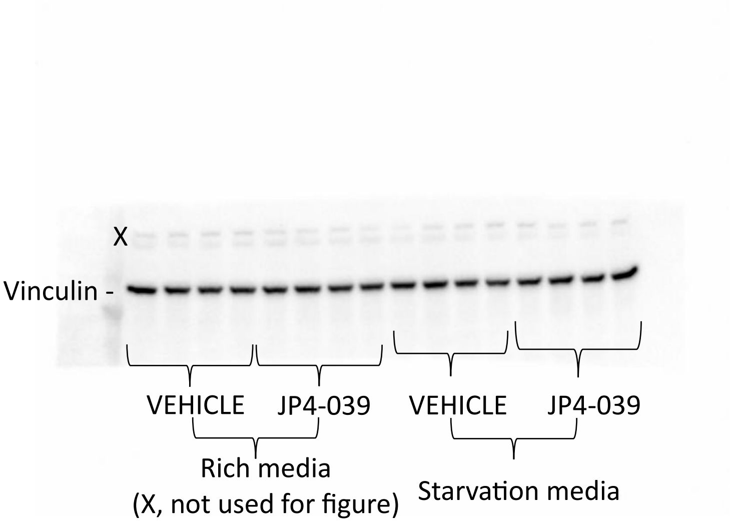

B – Original image showing Vinculin bands. **This is the image shown in Figure 5B for vinculin.** Bands marked with an X were not used for the figure.

Acquisition Information:

Exposure time: 2.556 seconds

Mode: Auto – Intense bands

Application: Chemi Hi Sensitivity

Excitation source: No illumination

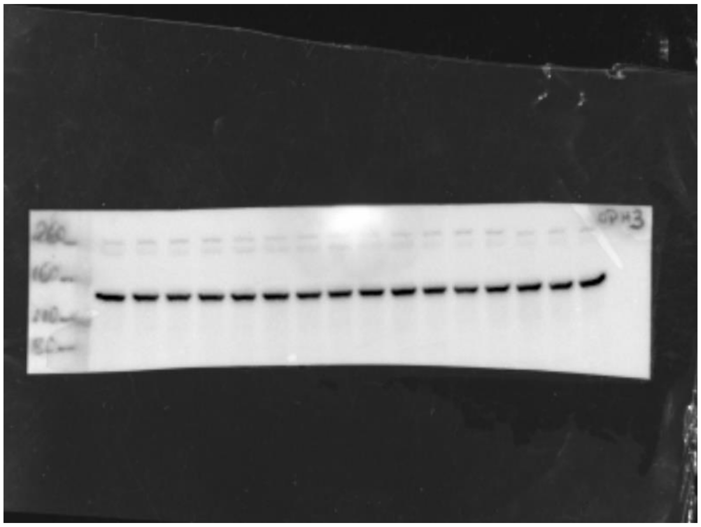

C – Merged image showing overlap of Vinculin bands and molecular weight marker.

All images shown in this file were acquired by Chemidoc MP from Biorad using the ECL (Enhanced chemiluminescence) method.
